# Threats to professional dignity of Iranian clinical nurses: A qualitative study

**DOI:** 10.1002/nop2.1492

**Published:** 2022-11-23

**Authors:** Ali Abbasi, Alice Khachian, Abbas Ebadi, Hosein Bagheri

**Affiliations:** ^1^ Department of Medical Surgical Nursing, Nursing Care Research Center, School of Nursing and Midwifery Iran University of Medical Sciences Tehran Iran; ^2^ Behavioral Sciences Research Center, Lifestyle Institute Baqiyatallah University of Medical Sciences Tehran Iran; ^3^ Nursing Faculty Baqiyatallah University of Medical Sciences Tehran Iran; ^4^ Department of Nursing, School of Nursing and Midwifery Shahroud University of Medical Sciences Shahroud Iran

**Keywords:** clinical nurse, content analysis, dignity, professional dignity, qualitative study

## Abstract

**Aim:**

Identifying threats to the nurses' professional dignity has an important role in maintaining and promoting their dignity. This study aimed to evaluate the perception of Iranian nurses' experiences of threats to their professional dignity in clinical settings.

**Design:**

A qualitative directed content analysis approach was used.

**Methods:**

The present qualitative study was conducted in Iran.Clinical nurses were selected using the purposive sampling method. Data were collected through in‐depth semi‐structured interviews with 15 clinical nurses from October 2020 to March 2021. The qualitative content analysis method was used to analyze the data.

**Results:**

Two main categories and 16 subcategories were extracted as follows: (1) professional factors (containing seven subcategories) and (2) organizational factors (containing nine subcategories).

**Conclusions:**

To promote the professional dignity of nurses, it is recommended to identify the factors threatening their professional dignity and create healthy work environments for them.

## 1 INTRODUCTION

Dignity is regarded as an indescribable and transcendent value (Leget, 2013) and should at all times be respected as it refers to their worthiness as human beings (Andorno, 2014; Badcott & Leget, 2013; Lindwall & Lohne, 2021; Milton, 2008). The concept of dignity can be classified into “absolute” and “relative” dignity. Absolute dignity is a universal value based on human rights and includes holiness, human worth, freedom, responsibility, duty and serving one's fellow man. All people are valuable, irrespective of their status or conditions, because they are human. Relative dignity on the other hand can change and is affected by culture, society and education (Edlund et al., 2013; Heijkenskjöld et al., 2010; Sandgren et al., 2021). Relative dignity is vulnerable, especially for nurses who are occasionally harassed, discriminated against and humiliated in the workplace (Khademi et al., 2012; Lin et al., 2011).

However, nurses' professional dignity is a relatively new concept (Combrinck et al., 2020; Joan Yalden & McCormack, 2010; Stievano et al., 2019). Stievano et al. (2012) defined the nursing professional dignity as a complex concept, including the individual's social and intrinsic characteristics. These characteristics are based on individual characteristics, inter‐ and intra‐professional relationships, workplace characteristics, professional competence and nurses' experience, public acceptance, and professional autonomy.

Professional dignity can be changed, threatened, or promoted through social communications (Stievano et al., 2012). Fowler (2018) mentioned that if all individuals have a worth and dignity that must be affirmed by the nurse, the nurse, too, has a worth and dignity that must be affirmed (Fowler, 2018). In fact, the professional dignity of nurses is strengthened when they are respected, appreciated and considered by others (Galuska et al., 2018). Parandeh et al. (2016) stated that many individual and organizational factors, including the attitudes of the community and the professional healthcare team are effective in developing or undermining the dignity of nurses.

### 1.1 Background

The results of various studies show that if the individuals' dignity is not preserved, it can lead to feelings of insecurity, guilt, worthlessness, despair, lack of self‐confidence, reduced quality of patient care, decreased satisfaction, increased desire to leave the profession and creation of the traditional image of nursing as a non‐professional occupation (Joan Yalden & McCormack, 2010; Khademi et al., 2012; Stievano et al., 2012). Hence, identifying threats to the professional dignity of nurses and paying attention to its promotion methods can enhance their professional structures and provide intuition, leading to improved patient‐based care (Combrinck et al., 2021).

Few studies have been conducted on threats to nurses' professional dignity. In this regard, Najafi et al. (2017) concluded that the dignity and professional reputation of nurses are seriously threatened by patients, their families, colleagues, superiors, and physicians, regardless of the type of violence. They categorized these threats into four categories: physical violence, psychological violence, honour insults and ethnic‐religious insults. Moreover, Khademi et al. (2012) identified the dignity violations as follows: “disrespect”, “coercion and violation of autonomy”, “ignoring professional and scientific capability” and “denying the value of nurse/care.” In this regard, Sabatino et al. (2016) mentioned that the low number of nurses, insufficient time and excessive working hours reduce the respect for nurses' dignity and professional values. The results of a study by Stievano et al. (2013) showed that nurses are dissatisfied with their work mainly due to a lack of respect for their professional dignity. They also emphasized the need for more research in other countries and different clinical settings better to understand the concept of nurse's professional dignity and its threatening factors.

Maintaining the professional dignity of nurses, as one of their fundamental rights, should be pursued as a moral necessity. Nurses are more prone to foster patients' dignity, patients' safety, and a better quality of care if their dignity is respected. Having more knowledge about the threats to professional dignity is a fundamental way to eliminate these threats and give a favourable work environment for nurses to provide ethical and respectful patient care. Therefore, this study was conducted to describe Iranian nurses' experiences about the threats to their professional dignity in clinical settings.

## 2 METHODS

### 2.1 Study design

Based on the study objectives, a directed qualitative content analysis approach was used in this qualitative research. The research question was: What are the Iranian clinical nurses' perceptions of threats to professional dignity?

### 2.2 Participants and setting

Using the purposive sampling method, which is used in qualitative studies to identify and select rich information for more efficient use of limited resources (Palinkas et al., 2015), this study was conducted on 15 Iranian nurses in clinical settings. The sampling continued until data saturation. The nurses were selected from four educational hospitals affiliated to Iran University of Medical Sciences and Shahroud University of Medical Sciences in Iran.

The inclusion criteria for nurses were: a bachelor's degree or higher; having at least 1 year of experience working in clinical settings; willingness to participate in the study and the ability to express the nurses' views about the intended concept (Table 1).

**TABLE 1:** Participants' main characteristics

Participant	Age (Years)	Gender	Marital status	Working departments	Degree of education	Work experience (years)
1	42	M	Married	Hemodialysis	MScN	20
2	28	F	Single	ICU	BSN	6
3	29	M	Married	ICU	BScN	6
4	30	M	Married	Emergency	BScN	7
5	43	M	Married	Emergency	MScN	20
6	26	F	Single	Emergency	BScN	3
7	37	M	Married	ICU	MScN	14
8	27	M	Single	Surgical	BScN	2
9	34	F	Married	NICU	MScN	10
10	29	F	Single	ICU	BScN	6
11	42	F	Married	Urology	BScN	19
12	36	F	Married	ICU	BScN	13
13	30	M	Married	ICU	BScN	7
14	29	F	Married	ICU	BScN	6
15	47	M	Married	Oncology	BScN	22

Abbreviations: BSCN, bachelor of science in nursing; F, female; ICU, intensive care unit; M, Man; MScN, master of science in nursing.

### 2.3 Data collection

We collected the data through conducting face‐to‐face in‐depth semi‐structured interviews with all the participants in a private and calm setting from October 2020 to March 2021. A pilot test was performed by conducting two interviews, based on which the process of main interviews and data analysis began. Social distance during the interview was at least 1.5 m with the interviewees. Open questions based on predetermined subcategories were used for interviews. The interview started with a general question: “Can you tell us about your own experiences about the nurses' professional dignity?” This question was asked in more detail: “What threatens your professional dignity?” Then interviews were followed by questions based on subcategories and probing questions. We used such open questions as “What do you mean by this?” “Please elaborate on it”, and “Please give an example” to obtain more proper answers. The interviews lasted 32–75 min (43 min on average). The transcribed text was returned to the contributors for approval. It is worth mentioning that two informed consent, one for agreement of participation and the second for agreement to the phonography, were taken from participants.

### 2.4 Data analysis

In parallel with data collection, the directed content analysis proposed by Hsieh and Shannon (2005) was used. The directed qualitative content analysis method is more structured than the conventional approach. According to this approach, the known key threats to the professional dignity of clinical nurses based on the literature review were considered the initial categories. In the next step, operational definitions of threats were extracted for each category. Data analysis began with repeated reading of the full texts of all the interviews. Then, the whole texts were read word to word and accurately. Codes were placed in pre‐identified categories based on conceptual similarity, and subcategory concepts were formed depending on the extent and logical relationships of the data in the categories. In this study, the data management (data storage) and qualitative data analysis (coding) were done through MAXQDA software.

### 2.5 Trustworthiness

The accuracy and stability of the data were assessed using Lincoln and Guba (1985). To ensure the validity of the data, the researchers allocated enough time to collect the data; the nurses were selected from different shifts and wards with different demographic characteristics; the data were analyzed with great accuracy; and other team members cross‐checked the findings. The research team members (including three nursing professors and two Ph.D. students) performed a critical review to verify the data's reliability. An attempt was made to avoid bias in the data analysis phase. Finally, a detailed descriptive report was prepared about the data collection, classification, and analysis.

### 2.6 Ethical considerations

This study was approved by the Ethics Committee of Iran University of Medical Sciences (Approval ID: IR.IUMS.REC.1399.810), Iran. The required permissions were also obtained from the selected hospitals. Before data collection, a written informed consent was obtained from all participants and they were assured about the anonymity, privacy and confidentiality of their data. To answer the participants' questions, the contact information of the first author was provided to the nurses. All participants were free to withdraw from the study at any stage. Also, the participants were assured about keeping their information confidential.

## 3 RESULTS

After data analysis, two main categories and 16 subcategories were extracted related to threats to the professional dignity of clinical nurses: (1) professional factors and (2) organizational factors. Each of these categories includes subcategories, which are summarized in Table 2.

**TABLE 2:** Theme, main categories and subcategories related to threats to professional dignity of clinical nurses.

Subcategories	Main categories	Theme
	Professional factors	Unethical behaviors
		Nonprofessional image
		Occupational violence
		Financial perspective toward the profession
		Unspecific task description
		Insufficient competence
		Ignoring professional and scientific capability
	Organizational factors	Low work experience
		Poor organizational communication
		Physician's dominance
		High workload
		Excessive paperwork
		Unsuitable financial situation
		Lack of facilities and equipment
		Lack of support
		Injustice

### 3.1 Professional factors

The professional factors are components in the nursing profession that threaten the nurses' professional dignity. Some components of the nursing profession were identified as threats to the professional dignity of clinical nurses. This category included seven subcategories: unethical behaviours, non‐professional image, occupational violence, financial perspective towards the profession, unspecific task description, insufficient competence, and ignoring professional and scientific capability.

#### 3.1.1 Unethical behaviours

Unethical behaviour affects professionalism in health care. Most importantly, unethical behaviour by nurses causes serious harm to patients as well as themselves. According to the participants, unfortunately, some nurses have inappropriate and aggressive behaviour with patients, and they do not respect the patients' families and even their colleagues. These factors can result in tension between the nurse and others. In this regard, one nurse mentioned:

Too often, nurses have inappropriate behavior with the patient or his/her family. For example, they tell them ‘I don't have time’, “why do you ask so many questions”, and ‘ask the doctor’; this causes them to be insulted, making the patients think that the nurses never have time or nurses are incompetent people or they have no morals at all. (P7)

#### 3.1.2 Non‐professional image

According to the nurses' experiences, most people consider nurses incompetent and harsh individuals, and they do not differentiate between them and the supporting staff; even many people consider those who take care of their children at home as nurses. This non‐professional image severely damages the professional dignity of nurses in society. In this regard, one of the nurses said:

Sometimes when I go to a group and say that I am a nurse, they used to tell me that you do injections, or you set up intravenous drips. They don't know what caring for a patient means at all. Sometimes they really confuse me with a medical secretary. They said that you are under the control of doctors; you do whatever the doctors order. (P10)

Another participant said:

The view of the community is very important; when the community still does not know the difference between a nurse assistant and a nurse and calls everyone a ‘nurse’, it is very annoying. (P7)

#### 3.1.3 Occupational violence

One of the most important threats to the nurse's professional dignity is the violence by patients and their families, nursing colleagues, nursing supervisors and physicians. Regarding the violence used by patients and their companions, one of the participants said:

Sometimes it happens that in times of crisis, especially when a patient expires, the patient's relatives come and even enter the ICU forcibly and start a riot, fighting, cursing, and insulting the nurses. Well, this damages the nurse's dignity. (P13)

Violence by physicians was a case mentioned by many participants:

We had a physician who would come and visit the patients. He always humiliated the nurses right from the very beginning of his ward visit, insulting them by saying, for example, ‘Why do you do this for my patient? I told you to do something else. (P2)

#### 3.1.4 Financial perspective towards the profession

Unfortunately, some nurses start the profession without any interest and continue working only to have a steady income. This financial perspective makes the nurses not take enough care of the patients as human being. If the materialistic view takes precedence over other fundamental values of the nursing profession, it will cause severe damage to the nurses' professional dignity. One of the participants said:

… a number of hospitals established some private wards only for COVID‐19 patients, which had a very good income and was welcomed by many nurses. They attended these shifts only for the sake of money, and practically didn't take enough care of the patients. They did not value the patients and just wanted to serve their own purpose, which was more financial gain. This devastated the value and dignity of patients and that of nurses, too. (P10)

#### 3.1.5 Unspecific task description

In most cases, the nurses' duties were changed according to the ideas of managers and physicians, and many of the responsibilities of physicians were assigned to nurses. Also, due to the lack of supporting staff, the nurses were forced to perform their duties along with their own ones. Unspecific task descriptions and forcing them to perform the duties of other professions damage the professional dignity of nurses. One of the participants said:

… it happens quite frequently that due to the absence of service staff, you have to do the chores that are beneath your level. In my opinion, nurses are professional staff, and doing the basic chore stops them from using their expertise. (P1)

#### 3.1.6 Insufficient competence

Many nurses even ignored the knowledge related to the department in which they worked, and only did practical work without relying on knowledge and without trying to update themselves scientifically. In fact, insufficient competence creates problems in patient care and tensions in communication with physicians, patients and patients' families, ultimately damaging the professional dignity of nurses. One of the participants said:

The nurse should seek to develop her own knowledge. When a patient asks you a question and you hesitate and say ‘I don't know’ and somehow elude them, they well realize that you are not reliable. (P2)

#### 3.1.7 Ignoring professional and scientific capability

Unfortunately, ignoring the value and role of nurses in the process of treating patients is induced in nurses through messages and feedback received directly or indirectly. As a result, it is an important factor in destroying their professional dignity. On the basis of the participants' experiences, most of the time, physicians, patients and their families undermine the scientific nature of the nursing profession. As one of the participants said:

… from the time emergency medicine specialists came, they no longer trust nurses in CPR and don't allow them to do even the routine chores. I feel like I'm not playing any role in the CPR process, and I'm not allowed to do anything without their permission; and this will surely have a bad effect on the dignity of nurses. (P1)

### 3.2 Organizational factors

The organizational factors threatening the nurses' professional dignity were related to the organizational characteristics. Some components of the organization, which were extracted based on the experiences of the participants, were identified as threats to the professional dignity of clinical nurses. These organizational factors are presented in the following sections.

This category included nine subcategories entitled low work experience, poor organizational communication, physician's dominance, high workload, excessive paperwork, unsuitable financial situation, lack of facilities and equipment, lack of support and injustice.

#### 3.2.1 Low work experience

The hospitals temporarily recruit nursing graduates to address the shortage of nursing staff. The lack of staff is the reason to allow novice and inexperienced nurses to start working right‐away without sufficient introductory training. On the other hand, some of the nursing managers have high expectations from these nurses. Also, the employment of low experience nurses in the intensive care unit and emergency departments causes many problems. Therefore, the nurses are dissatisfied, and their professional dignity is damaged. In this regard, two participants said:

Honestly, over the first few months that I started my job, I was stressed a lot… there were a lot of working shifts Sometimes because I didn't want my colleagues to consider me a novice, I tried to ask them as few questions as possible and barely asked for help. After a while, I realized that I might make a mistake that would cause harm to the patient. Those days were so hard for me, and I even wanted to quit my job. (P2)

I think a new nurse does not know how to manage her/his time at work; s/he is perplexed. It makes those who are around to say: ‘Poor guy! S/he has always undone tasks.’ It can tarnish that nurse's dignity. (P3)

#### 3.2.2 Poor organizational communication

Unfortunately, one of the problems in some organizations is the poor communication skills of people with each other, which can also be witnessed in nurses and lead to many problems for them. Nurses' weak communication is an important barrier to inter‐professional and intra‐professional interactions and communication with patients and their families. One of the nurses said:

When we have difficulty communicating with others, it can have many consequences for the nurses, including the verbal insults and physical attacks from others, which can ultimately damage the nurse's professional dignity severely. (P15)

#### 3.2.3 Physician's dominance

The experiences of all participants showed the physician's dominance in the medical centers. In such organizations, physicians occupy the highest position and can make all the existing decisions, undermining nurses' competence and independent decisions. In this regard, one nurse said:

There was a physician who sometimes made mistakes himself and unfortunately acted in such a way as it was a nursing mistake. In such cases, the nurses cannot defend themselves, because the top managers in the system are physicians who decide in favor of physicians and support them; therefore, the ruined party is always the nurse. (P9)

#### 3.2.4 High workload

The insufficient nursing staff is an important issue in Iran, significantly increasing nurses' workload. Also, the workload of nurses dramatically increased during the COVID‐19 pandemic. On the basis of the participants' experiences, this factor not only reduced the quality of patient care but also threatened the professional dignity of the nurse. A participant stated:

The high workload has caused the patient care to be done with delay or not done at all. I want to tell you that when the work pressure is high, it is very difficult for the nurse to prioritize her tasks in a short time and to do it carefully. These will ultimately harm the patient care and can pose a challenge to the nurse's performance by others. (P12)

#### 3.2.5 Excessive paperwork

Most participants believed that a lot of paperwork in the nursing system kills the time to provide services to the patient and most of the nurse's time is spent on such paperwork. This reduces the quality of nursing care and severely threatens the nurse's professional dignity due to the negative reaction from managers, patients and families. Accordingly, one of the participants said:

When there is so much useless paperwork, how can a nurse find time to take care of the patient? Patient care is completely forgotten here. Unfortunately, the managerial system considers someone as a better nurse if she does better documentation and paperwork and the quality of patient care is not considered at all. These put the dignity of nursing at risk. (P10)

#### 3.2.6 Unsuitable financial situation

On the basis of the participants' experiences, financial concerns have created many problems for nurses about job and family affairs. The high workload, inadequate salaries, and delayed payments could demotivate the nurses and threaten their professional dignity. One of the nurses said:

… our hospital did not pay for our extra work for eight months; the nurses were really dissatisfied. Nurses take overtime shifts to get more money. When you are financially concerned, your dignity is damaged. (P6)

#### 3.2.7 Lack of facilities and equipment

Most participants believed that as the workplace is their second place of residence and they spend many hours there, inadequate facilities not only makes it difficult for nurses to perform their duties, but also poses an important threat to the professional dignity of nurses. One of the participants said:

One of the problems that most nurses have is their small resting area, which also serves as their dining room and creates much difficulty for them. We spend so many hours in the hospital and the ward and expect to have the minimum favorable facilities so that we can work more efficiently. But when this doesn't happen, we naturally feel that we are not valued, so, our dignity is undermined. (P14)

#### 3.2.8 Lack of support

Unfortunately, in many cases, the participants' experiences showed that in difficulties, as well as financial and emotional crises, the managers do not do much and they ignore nurses. This factor was mentioned as one of the most important factors for damages to the nurses' professional dignity. In this regard, one of the participants said:

We had a head nurse who was not rational at all and did not pay attention to the nurses' problems. For example, one day, he would say that you must come tomorrow on an unplanned shift. No matter how much I explained that I had a lot of things planned to do in my day off; he would answer that ‘you must come’. These situations really tarnished my dignity. (P8)

#### 3.2.9 Injustice

One of the important issues threatening the professional dignity of nurses is discrimination and organizational injustice. This injustice was apparent in discriminated payments, facilities and equipment, arrangement of shifts, etc. One of the participants said:

… the per‐service payment regulation has not been set for nurses yet, and there is no real difference between a nurse who does her best and a nurse who doesn't care. Why should then we work well for an organization that does not value us at all and there is so much discrimination? These problems negatively affect the nurse's dignity. (P11)

## 4 DISCUSSION

In this study, according to the participants' experiences, the threats to the professional dignity of clinical nurses were placed in two categories of professional and organizational factors.

The professional factors included items related to the nursing profession. One of the threats to the nurses' professional dignity was unethical behaviour of nurses. In this regard, Sabatino et al. (2016) stated that the unethical behaviours of some nurses in interaction with others play an important role in undermining their professional dignity. Valizadeh et al. (2018) also considered passive behaviours and inattention to patients threatening the nurses' professional dignity.

One of the main threats to the nurses' professional dignity was the nonprofessional image. In this regard, various studies have shown that social stereotypes, such as the nonprofessional and traditional nursing image, negatively affect nurses' dignity (Khademi et al., 2012; Lawless & Moss, 2007; Valizadeh et al., 2018). Comparing physicians and nurses, Heshmati Nabavi et al. (2014) stated that nurses have less credibility than physicians in healthcare systems and a negative attitude exists towards nursing.

The results of this study showed that occupational violence was one of the most important threats to the professional dignity of nurses. Several studies have shown disrespect and humiliation of health professionals, especially nurses in the workplace (Arnetz et al., 2018; Bambi et al., 2017; Najafi et al., 2017). The violence by patients, their families, colleagues, superior nurses or physicians (Najafi et al., 2017). Humiliation and disregard are considered as factors threatening the nurses' professional dignity (Khademi et al., 2012).

One of the problems of the nursing profession is the financial perspective towards the profession. Sometimes, due to the relatively low income and the increasing cost of living, the nurses increase their work shifts to meet their financial needs. As a result, they may neglect some safe nursing care for patients, undermining the professional dignity of nurses. Kalandyk and Penar‐Zadarko (2013) stated that the main cause of dissatisfaction and disruption in nursing care is low income. On the other hand, if the materialistic view takes precedence over other basic values of the nursing profession, it will seriously damage the nurses' professional dignity. The results of a study by Dos Santos (2020) showed that if financial considerations in the nursing profession become a nurse's priority, they may affect other nursing values and damage professional dignity.

Unspecific nurses' tasks were one of the major threats to clinical nurses' professional dignity. In this regard, Sabatino et al. (2016) stated that confusion in the activities between nurses and support staff plays a crucial role in undermining the nurses' professional dignity. Also, asking nurses to perform secretarial actions is a case that occurs due to physicians neglecting nurses' tasks description. Grosso et al. (2019) stated that overexposure to the duties and expectations of nurses outside the professional roles of nurses will damage their professional dignity.

The results of this study showed that insufficient competence is one of the most important threats to nurses' professional dignity. The importance of nurses' knowledge and professional competence was demonstrated in various studies. When nurses use sufficient knowledge and skills in patient care, they experience patient appreciation and respect from colleagues (Combrinck et al., 2020; Halverson, 2020; Siles‐Gonzalez & Solano‐Ruiz, 2016; Valizadeh et al., 2018). In this regard, Hanifi et al. (2012) stated that knowledge increases the sense of worthiness, credibility, competence, professional power and the promotion of dignity. On the other hand, insufficient competence creates many problems for nurses, and ultimately damages their professional dignity.

According to our results, another threat to the nurses' professional dignity was ignoring the professional and scientific capabilities. In this regard, Sabatino et al. (2014) stated that ignoring nurses' professional and scientific capabilities is an important factor threatening professional dignity. Moreover, according to some novice nurses, constant evaluation by managers implies a kind of distrust, ignoring their knowledge and skills. Meanwhile, experienced nurses find the lack of participation in the decision‐making process a disregard for their professional abilities, causing them to seek revenge on the managers and system (Khademi et al., 2012). On the other hand, in interprofessional relationships, when physicians ignore nurses' abilities, nurses' professional dignity is severely damaged (Joan Yalden & McCormack, 2010; Stievano et al., 2012).

Organizational factors included threats that existed in obsolete organizational models. In this regard, one of the nurses' professional dignity was the low work experience of nurses. The employment of inexperienced nurses in the emergency ward and ICU along with the high expectations of managers from them at the beginning of their work led to their fatigue and frustration, damaging their professional dignity. In this regard, the results of the study by Khademi et al. (2012) showed that inexperienced nurses have witnessed a lot of pressure from senior managers and have reported many problems such as threatening and insecure working atmosphere, lack of right in making decisions and compulsion. Communication, a crucial element in providing high‐quality health care services, leads to patient satisfaction and health. Unfortunately, the inability of some nurses to communicate well in organization leads to disrespect from patients and their families, colleagues, physicians, and other health care professionals, threatening the clinical nurses' professional dignity. In this regard, Sabatino et al. (2016) stated that inefficient communication with physicians and others reduces professional dignity. Lawless and Moss (2007) stated that although nurses can use strategies such as having a positive attitude and strong relationships with others to support their dignity, these strategies are less evident in nurses' experiences.

According to our results, the physician's dominance was one of the major threats to the clinical nurses' professional dignity. Various studies have mentioned the dominance of the medical model as an important threat (Khademi et al., 2012; Sabatino et al., 2014; Stievano et al., 2012; Valizadeh et al., 2018). The physicians' superiority is evident in hospitals and the public idea considers the physicians as the main role players because physicians make the main decisions about patients (Valizadeh et al., 2018). Another organizational factor was the high workload. High workload are the most important threats to the nurses' professional dignity, which will divert them from their main task (Dwyer et al., 2009; Lawless & Moss, 2007; Sabatino et al., 2016; Valizadeh et al., 2018). In this regard, many nurses have experienced a lack of respect for their dignity due to the high workload. As a result, they have been forced to change their departments or even quit their job (Valizadeh et al., 2018). Also excessive paperwork is one of the organizational factors that can lead to negligence or inhibition of the expression of nurses' competencies and damage to their professional dignity (Khademi et al., 2012; Stievano et al., 2012). Sabatino et al. (2016) stated that filling too many documents is one of the features of obsolete organizational models. Also, another threat to the nurses' professional dignity was the unsuitable financial situation of nurses. When nurses do not have sufficient income, work and life problems increase, and the nurse's focus on providing high‐quality nursing care decreases. Ultimately, this will be an important factor in damaging the professional dignity (Sabatino et al., 2014; Sturm & Dellert, 2016).

The lack of facilities and equipment was another threat to the professional dignity of nurses. Khademi et al. (2012) stated that nurses experienced disrespect due to the lack of facilities and equipment in their workplaces. In addition, the poor quality of such services and facilities can lead to humiliation. The lack of support threatened the clinical nurses' professional dignity. Various studies have shown that the managers' lack of attention and support has an important role in undermining the nurses' professional dignity (Dwyer et al., 2009; Najafi et al., 2017; Valizadeh et al., 2018). According to Valizadeh et al. (2018) such feelings as emptiness, worthlessness, and discouragement are created among nurses due to the lack of support from managers and head nurses. Finally, injustice was another factor threatening the professional dignity of nurses. Various studies showed nurses' discrimination and inequality with other healthcare team members (Joan Yalden & McCormack, 2010; Khademi et al., 2012). According to Najafi et al. (2017), most nurses were dissatisfied with the managers' discriminatory behaviours and their unlimited support for patients, families and physicians. In addition, most nurses complained of payment discrimination (Joan Yalden & McCormack, 2010).

### 4.1 Limitations

This study had some limitations. First, data collection was carried out in Iran and included only two cities. Hence, the factors threatening the professional dignity of nurses in other cities and outside Iran may be different. Second, the concept of nurses' professional dignity was based on the intellectual views of the participants in a qualitative study. So, using other research approaches could reveal more information about this concept.

### 4.2 Implications for nursing

Identifying the factors threatening the professional dignity of nurses can play a significant role in improving the awareness of the nursing managers and organizations. As a result, they can apply various interventions to eliminate threats and create healthy work environments.

## 5 CONCLUSIONS

The findings of this research showed the factors affecting the dignity of nursing. On the basis of the findings, considering the threats to nurses' professional dignity is useful for achieving the goals of nurses and organizations. The present study's findings showed that the threats to clinical nurses' professional dignity fall into two categories of professional and organizational factors. Identifying and nullifying these factors to improve the nurses' working environment and quality of life can promote their professional dignity and enhance the quality of care. When the dignity of nursing is threatened, there will be an inability to perform, so the issue of nursing dignity is an important issue that must be paid attention to increase the efficiency and performance of nurses. It is recommended to use the findings of this research to plan and implement applied research to develop and implement it in the hospital and nursing management.

## AUTHOR CONTRIBUTIONS

Study design: Ali Abbasi, Alice Khachian, Abbas Ebadi, Hosein Bagheri. Data collection: Ali Abbasi. Data analysis: Ali Abbasi, Alice Khachian. Abbas Ebadi. Study supervision: Alic Khachian. Abbas Ebadi. Manuscript writing: Ali Abbasi, Alice Khachian, Abbas Ebadi, Hosein Bagheri. Critical revisions for important intellectual content: Alice Khachian, Abbas Ebadi.

All authors have agreed on the final version and meet at least one of the following criteria [recommended by the ICMJE (http://www.icmje.org/recommendations/)]:

- Substantial contributions to conception and design, acquisition of data or analysis and interpretation of data;
- Drafting the article or revising it critically for important intellectual content.

## FUNDING INFORMATION

This research received no specific grant from any funding agency in the public, commercial, or not‐for‐profit sectors.

## CONFLICT OF INTEREST

No conflict of interest has been declared by the author(s).

## ETHICAL APPROVAL

The Ethics Committee of Iran University of Medical Sciences, Tehran, Iran, approved this study (code: IR.IUMS.REC.1399.810).

## Data Availability

Data related to this study can be availed on request from the first author of this manuscript.
